# Supplementary material for: hLMSC Secretome Affects Macrophage Activity Differentially Depending on Lung-Mimetic Environments
Source: Cells. 2022 Jun 8;11(12):1866. doi: 10.3390/cells11121866 (PMC9221297; doi:10.3390/cells11121866)

Supplement Material and methods

1. Materials and Methods

Unless otherwise specified, reagents for cell culture were purchased to Thermo Fisher Scientific, Massachusetts, USA and the reagents employed for non-cellular protocols were purchased to Sigma-Aldrich, St Louis, USA.

Patient material

Institutional Review Board Statement: The study was conducted according to the guidelines of the Declaration of Helsinki and approved by the Ethics Committee of Lund (Dnr 2015-891, 2015-12-21), Gothenburg (Dnr. 413/2008, 2008-08-19), Sweden. Informed consent was obtained from all subjects involved in the study.

Isolation of hLMSC

Adult lung-derived MSC were isolated from peripheral transbronchial (parenchymal tissue) biopsies from 6 different donors (main details are specified in Table 1) as described elsewhere (182). Briefly, lung biopsies were cut into small pieces, washed and cells were isolated by enzymatically digestion with collagenase type I, 300 U/mL, hyaluronidase, 1 mg/mL and DNAse in Dulbecco's phosphate buffered saline (DPBS). After digestion, cells were washed and seeded adherent on standard plastic tissue flasks with StemMACS MSC Expansion medium (Miltenyi Biotec, Bergisch Gladbach, Germany) supplemented with 10 % FBS (Hyclone Laboratories, Logan, USA) and 1 % Antibiotic-Antimycotic. Medium was changed weekly and cells were passaged with TrypLE Express at 70–90% confluence. After passage 2, hLMSC medium formulation was changed to DMEM supplemented with FBS (10%) to expand the cells prior to the experiments. Cells were used between passage 4 and 7.

Preparation of the lung scaffolds

Lung scaffolds were obtained from pig lungs, tissue cubes of ~ 1x1x1 cm^3 were 350-µm sliced and decellularised with a protocol described elsewhere (50). Briefly, the slices were incubated in a decellularization solution containing 8 mM CHAPS, 1 M NaCl, and 25 mM EDTA in PBS. The incubation was performed in motion with 1 ml/slice of decellularization solution, which was changed six times over a period of four hours at room temperature. Then, slices were submerged in benzonase working buffer (20 mM Tris-HCl, 2 mM Mg^{2+} and 20 mM NaCl at pH 8) , and incubated for 30 min at 37 °C. Decellularized slices were rinsed in PBS and stored until use at +4 °C in PBS containing 1% of Antibiotics.

Preparation of the lung-derived hydrogels

ECM hydrogels were prepared from porcine lung tissue from 3 different animals obtained from a local slaughterhouse. The lung tissue was first decellularized by using an adapted protocol from Pouliot, et al. (168), where the lungs were perfused through the trachea and the vasculature with 0.1% Triton X-100, sodium deoxycholate, DNase and 1 M sodium chloride, with intermediate perfusion with distilled water and PBS for rinsing purposes. Afterwards, decellularized lungs were cut into small pieces and frozen at -80 °C, freeze-dried (Telstar Lyoquest-55 Plus, Terrassa, Spain), and pulverized into micron-sized particles at -180 °C by using a cryogenic mill (SPEX 6755, NJ, US) for 5 min at maximum speed. The resulting powder was digested at a concentration of 20 mg/ml in a 0.01 M HCl solution with pepsin from porcine gastric mucosa (1:10 concentration) under magnetic stirring at room temperature for 16 h. Pregel solution was then pH-stabilized to 7.4 (±0.4) by using 0.1 M NaOH, added PBS 10X and frozen at -80 °C for subsequent use.

Stretch device and functionalization

To subject the cells to stretch, a custom-made device was built based on a previous design described elsewhere (Campillo N 2016). The device consists of 3 wells made of two polydimethylsiloxane (PDMS) layers separated by a commercially-available PDMS membrane (Gel-Pak, Hayward, CA) of 150 µm. Membranes were deflected to subject cells to ~20% of circumferential stretch at a frequency

of 0.2 Hz. DLS, hydrogels and well-plates with a PDMS membrane attached (as a plastic control) were repopulated with hLMSC and subjected or not to stretch. In order to promote either cell adhesion or attachment of DLS and hydrogel (Figure 1A), the PDMS membranes were functionalized with collagen I. Briefly, the membranes were plasma-irradiated during 1 minute with a hand-held corona BD-20AC (Electro-Technic Products, Chicago, USA), treated with 10% APTES during an hour, rinsed and treated with 5% genipin for 45 minutes. After final rinses, membranes were dried overnight. Next day, membranes were UV-sterilized for 15 minutes and incubated with a collagen I solution (0.3 mg/ml) during at least 2 hours and washed to remove excess solution prior to either cell seeding of attachment of DLS and hydrogels. The DLS and hydrogels were placed onto the membrane and allowed to attach overnight #GenchiGetal. Colloids Surf B Biointerfaces 2013

Physiomimetic culture of hLMSC

DLS were repopulated with a concentrated suspension of hLMSC (40000 cells/cm^2), incubated for 1 hour prior to adding 2 ml of culture medium and then incubated for another hour under mild agitation (60 rpm). Hydrogels were formed by mixing the pre-gel solution at with a cell amount to obtain a mixture of 400000 cells/cm^3 and then poured gently onto the treated wells. Cell-laden hydrogels were crosslinked at 37° C in the incubator during 1 hour prior to adding medium. The plastic controls were seeded with 8000 cells/cm^2. The seeded cells for all conditions were incubated for 24 hours in DMEM containing 10% FBS prior to starting the mechanical stimulation. The medium was changed for DMEM containing 2% and the stretch devices were connected to the mechanical stimulation (CS) for 96 hours with daily changes of medium (DMEM with 2% FBS) for all conditions (Fig 1B). The final 24 hours the cells were cultured in DMEM without FBS. The experiment was repeated with 6 different healthy donors and were assayed in triplicates.

At the endpoint, the conditioned medium (CM) from all groups was collected and centrifuged 1000g for 5 minutes at 4ºC and stored at -80. The repopulated DLS and hydrogels were either saved for histological evaluation or RNA analysis.

Confocal immunofluorescence.

Repopulated L-Scaffolds and L-Hydrogels were fixed in 4% formaldehyde for 45 minutes at room temperature and stored in PBS with 0.05% sodium azide (Sig-ma-Aldrich) at 4°C until further processing. This included permeabilization in 0.1% Triton X-100 (Sigma-Aldrich) for 5 minutes at room temperature and staining with 1X Phalloidin-iFluor 555 (Abcam, Cambridge, UK) for F-actin staining and DAPI (nuclei staining) prepared in 1% BSA in PBS for 1 h at room temperature. The L-Scaffolds and L-Hydrogels were immersed in Ce3D (prepared as previously described[20]) in cham-bers built on top of slides using iSpacers (SunJin Lab) and glass coverslips. They were imaged in a resonant scanner A1RHD confocal microscope (Nikon) controlled with the NIS Elements AR software (Nikon) using a 10X air objective (Nikon) and laser excita-tion. Images were corrected for brightness and prepared for publication in NIS Ele-ments AR Analysis software (Nikon)..

Multiplex secretome evaluation

Supernatants were assayed for 13 markers by a multiplexed ELISA custom-made plate (R&D, Minneapolis) that detected the following proteins: IL-1ra, m-CSF, MCP-1, MIP-2, IL-1β, IL-6, IL-8, and TNF-α, IFN-γ IFN S γ-10, VEGF, IL-4 and HGF. The data was quantified as net median fluorescence intensity according to a MAGPIX 2uminex reader (Luminex, Austin, USA). The data was normalized to cell number and UMAP plots were created in Rstudio (2021.09.1 based on R 4.1.2) with the umap package (0.2.7.0) and ggplot2 (3.3.5). The other graphs were made using GraphPad Prism (9.3.1). The vinyl graphs are plotted as parts of whole and thus normalized in between each group.

Phagocytosis assay

Human monocytes (THP-1, TIB-202, ATCC) were expanded in suspension using RPMI 1640 medium (ATCC reformulation) supplemented with 10% of FBS, 5mM of β-mercaptoethanol and 1% of Antibiotics, according to the manufacturer's protocol. When desired cells amounts were achieved, THP-1 cells were then differentiated into adherent macrophages by seeding 750000 cells/cm^2 in a 96-wells plate with a black bottom and cultured in fresh medium supplemented with 50 ng/mL of phorbol 12-myristate 13-acetate (PMA) and cultured during 48h. Phagocytosis was measured as described by the

Vybrant Phagocytosis assay kit. Briefly, the culture medium was removed and equal amounts of fluorescence particles were mixed with the CM from all culture conditions and added to the wells. After 3 hours of incubation, excess particles were removed and quenched within the well by trypan blue. The net intake of particles was quantified according to the fluorescence readout of the test samples subtracted the acellular controls and related to a positive control without the addition of CM from MSC.

Phenotype assessment of Macrophages exposed to MSC secretome
In another set of experiments, differentiated macrophages were polarized to M1/M2 phenotypes by the addition to the medium of either LPS (50 ng/ml) or with IL-4 (20ng/ml) and IL-10 (10ng/ml) (*R&D Systems, Minneapolis, USA)*. To assess the effect of the CM from the hLMSC cultures (hydrogels, scaffolds and plastic with or without CS), the CM was mixed simultaneously at 1:1 ratios with the medium of the M1 and M2 macrophages. M0, M1 and M2 controls (without mixing with CM) were cultured parallel to the other samples. After 24h of culture, cells were prepared for cytometry. Cells were recorded using flow cytometry on a BD LSRFortessa X20 and analyzed using FlowJo (10.8.1). Samples were clean in FlowJo using the FlowAI and then gated on FMO controls. A complete gating strategy can be found in supplement Figure S1. Antibodies were titrated for optimal performance starting at 1µg test or manufacture recommended concentration and used as following. CD11c (BV650, 563403 BD) were diluted 8 times for 0.625 µl per sample, CD40 (PE-Cy7, 561215 BD) were diluted 16 times for 0.313 µl per sample, CD80 (BV711, 751726 BD) were diluted 8 times for 0.625 µl per sample, CD86 (R718, 751920 BD) were diluted 8 times for 0.625 µl per sample, CD124 (BB700, 745925 BD) were diluted 3 times for 1.7 µl per sample, CD163 (BV786, 741003 BD) were diluted 4 times for 1.25 µl per sample, CD200R (BV421, 566344 BD) were diluted 8 times for 0.625 µl per sample, CD206 (APC, 550889 BD) were diluted 8 times for 2.5 µl per sample, CD281 (PE, 12-9911-42 eBioscience) were diluted 8 times for 0.625 µl per sample, HLA-DR (BB515, 564516 BD) were diluted 8 times for 0.625 µl per sample and two were used 7AAD (7AAD, 555816 BD) were diluted 2 times for 10 µl per sample and 7AAD (7AAD, A9400 Sigma) were diluted 200 times for 0.3 µl per sample. Two 7AAD was used due to availability but separate FMO controls were used for gating. All antibody samples were diluted in Brilliant stain buffer , 50 µl (563794 BD).

qPCR for mechanosensory markers
Cell-laden hydrogels and repopulated scaffolds were soaked in RNA later (Thermo Fisher Scientific, Massachusetts) to preserve RNA integrity until further use. RNA from MSC physiomimetically cultured was extracted using the mirVana RNA isolation kit later (Thermo Fisher Scientific, Massachusetts) and quantified the total RNA. The samples cultured directly onto the bare plastic were isolated the RNA using the RNeasy Mini Kit later (Thermo Fisher Scientific, Massachusetts). Afterwards, all samples were retro-transcribed using the Quantitect Reverse Transcription Kit. Changes in gene expression of the connective tissue growth factor (CTGF) and the Cysteine-rich angiogenic inducer 61 (CYR61) were quantified by quantitative PCR using the QuantiFast SYBR Green Master Mix in a StepOne Plus thermocycler. Relative CTGF and CYR61 was normalized to the expression of peptidylprolyl isomerase A (PPIA) applying the $2^{-\Delta\Delta Ct}$ method (183) to quantify the fold-change in expression of these genes compared to the plastic-ST group. All reagents for nucleic acid protocols were purchased to Qiagen, unless otherwise specified.

Statistical analysis
Data was presented as the individual values for each output where bar represents means. Differences among groups were analyzed by two-way repeated measurements analysis of variances (ANOVA) for the parameters of **environment** (Plastic, L-Scaffold and L-Hydrogel) and **strain** (static-ST or stretch-CS). The specific differences within the environments were assessed by non-parametric paired t-test (Wilcoxon). All statistical analysis were performed with GraphPad Software (San Diego, CA), considering $p<0.05$ as statistical significant.

Table S1. Characteristics of patients

Sample	Gender	Age	Status of the tissue	Tobacco (packets per year)	Tissue location	Other observations
Donor 2	Male	43	Healthy	Former smoker	Peripheral	Litium medication
Donor 3	Female	44	Healthy	Current smoker(31)	Peripheral	
Donor 4	Male	46-68	Healthy	Never smoker	Peripheral	
Donor 5	Female	74	Healthy	Former smoker	Peripheral	Adenocarcinoma and hypertension
Donor 6	Female	59	Healthy	Former smoker(34)	Peripheral	Adenocarcinoma and Sjögren´s syndrome
Donor 7	Female	81	Healthy	Former smoker (28)	Peripheral	Adenocarcinoma

Figure S1. Sorting FACS strategy

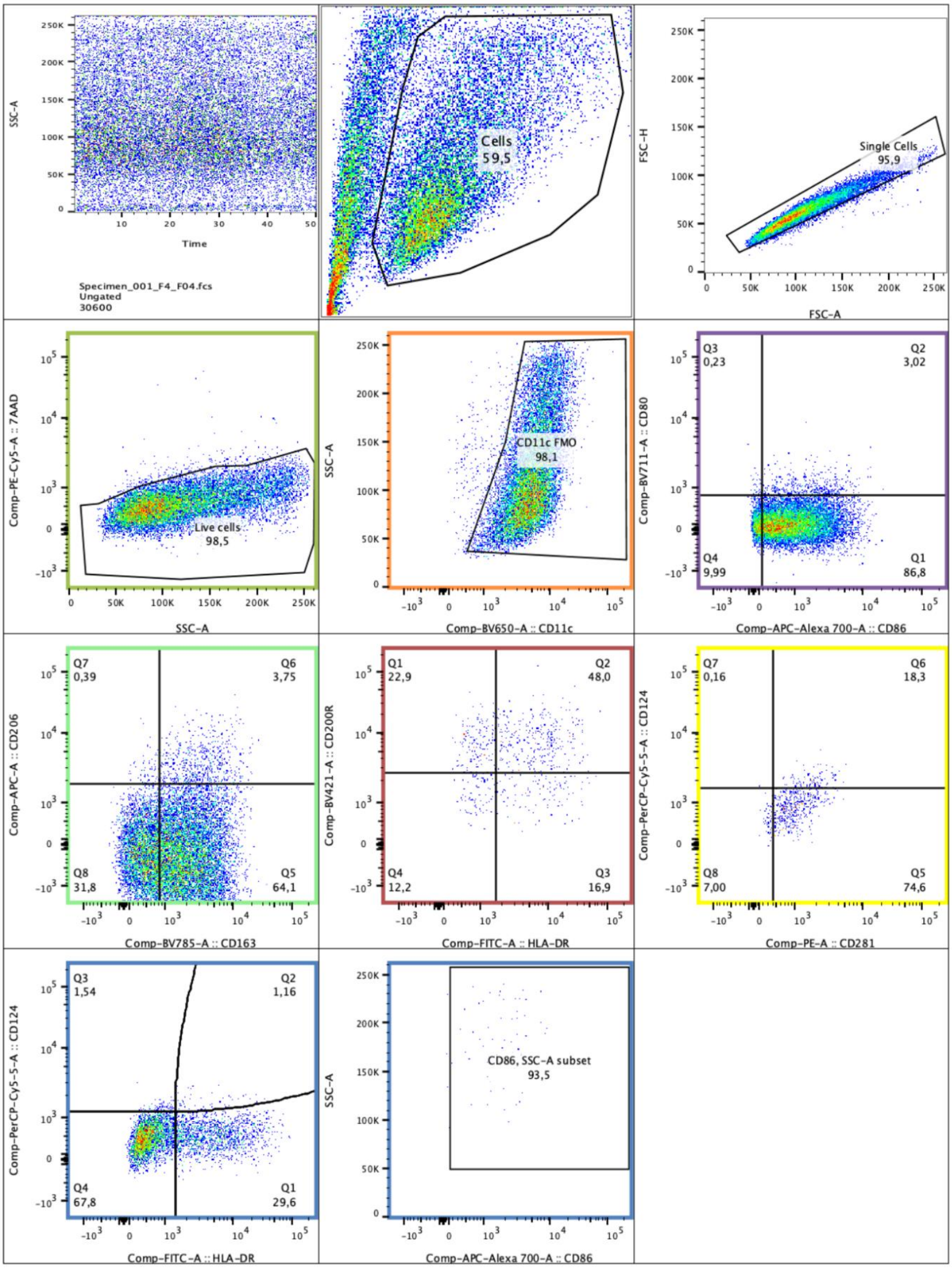

Table S2. % Live Macrophage subpopulations

Marker	Strain	Enviroment	Patient 3	Patient 6	Patient 7	Patient 2	Patient 4	Patient 5
% of live cells M1	Static	M0 Plastic	12,3	5,13	2,69	5,22	2,59	1,99
% of live cells M1	Static	M0 L-Scaffold	5,68	8,8	4,5	7,19	1,38	2,35
% of live cells M1	Static	M0 L-Hydrogel	5,98	8,9	3,52	5,12	2,83	0,53
% of live cells M1	Static	M1 Plastic	72,6	62,5	70,8	38,4	47,4	36,9
% of live cells M1	Static	M1 L-Scaffold	75,7	43,3	51	36		35,3
% of live cells M1	Static	M1 L-Hydrogel	61,2	72,7	45	34,1	44,3	41,1
% of live cells M1	Static	M2 Plastic	6,24	19,2	2,97	4,06	4,25	4,89
% of live cells M1	Static	M2 L-Scaffold	24,6	11,5	6,87	6,58	8,44	4,43
% of live cells M1	Static	M2 L-Hydrogel	11,6	6,08	7,05	9,19	3,19	4,91
% of live cells M1	Cyclic Stretch	M0 Plastic	8,73	7,17	4,59	2,69	1,32	3,78
% of live cells M1	Cyclic Stretch	M0 L-Scaffold	5,79	10,6	4,86	5,04	3,71	1,36
% of live cells M1	Cyclic Stretch	M0 L-Hydrogel	4,04	4,13	8,51	3,14	3,41	0
% of live cells M1	Cyclic Stretch	M1 Plastic	72,6	60	72,4	35,5	13,3	34,5
% of live cells M1	Cyclic Stretch	M1 L-Scaffold	73,5	31	46,7	23,2	18,4	36,4
% of live cells M1	Cyclic Stretch	M1 L-Hydrogel	55,3	66,9	61	40,6	40,5	28,4
% of live cells M1	Cyclic Stretch	M2 Plastic	8,27	13,9	8,43	4,42	5,06	2,97
% of live cells M1	Cyclic Stretch	M2 L-Scaffold	9,42	13	4,06	4,63	6,41	5,79
% of live cells M1	Cyclic Stretch	M2 L-Hydrogel	22	3,68	9,73	4,27	3,29	9,71
% of live cells M2a	Static	M0 Plastic	0,085	0	0,041	0,047	0,00832	0,00712
% of live cells M2a	Static	M0 L-Scaffold	0	0,092	0	0	0	0
% of live cells M2a	Static	M0 L-Hydrogel	0	0	0	0	0,016	0
% of live cells M2a	Static	M1 Plastic	0	1,18	0	0,58	0	0
% of live cells M2a	Static	M1 L-Scaffold	0,026	1,98	3,08	0	0	0
% of live cells M2a	Static	M1 L-Hydrogel	0,76	0	3,99	0	0	0
% of live cells M2a	Static	M2 Plastic	3,86	7,78	2,83	1,34	3,04	2,32
% of live cells M2a	Static	M2 L-Scaffold	15,6	6,96	8,03	0,4	2,32	2,81
% of live cells M2a	Static	M2 L-Hydrogel	8,51	1,31	9,4	0,52	3,26	2,58
% of live cells M2a	Cyclic Stretch	M0 Plastic	0,022	0,17	0,00768	0	0	0,013
% of live cells M2a	Cyclic Stretch	M0 L-Scaffold	0	0,19	0,01	0	0,042	0

% of live cells M2a	Cyclic Stretch	M0 L-Hydrogel	0,18	0	0,084	0	0	0
% of live cells M2a	Cyclic Stretch	M1 Plastic	0	0,22	0	0	0	0
% of live cells M2a	Cyclic Stretch	M1 L-Scaffold	0	3,03	3,75	0	0,23	0
% of live cells M2a	Cyclic Stretch	M1 L-Hydrogel	1,41	0	1,54	0	0	0
% of live cells M2a	Cyclic Stretch	M2 Plastic	5,1	6,06	7,2	0,7	1,52	1,76
% of live cells M2a	Cyclic Stretch	M2 L-Scaffold	6,32	5,7	3,68	0,22	2,36	2,65
% of live cells M2a	Cyclic Stretch	M2 L-Hydrogel	3,5	2,61	5,47	0,42	1,55	1,97
% of live cells M2b	Static	M0 Plastic	0,9	1,41	0,87	1,99	0,49	0,58
% of live cells M2b	Static	M0 L-Scaffold	0,39	0,84	0,4	2,69	0,4	0,4
% of live cells M2b	Static	M0 L-Hydrogel	0,73	1,24	0,62	0	0,88	0,32
% of live cells M2b	Static	M1 Plastic	3,16	2,11	1,43	2,91	0	0,49
% of live cells M2b	Static	M1 L-Scaffold	1,82	1,64	2,95	0,9	0	1,1
% of live cells M2b	Static	M1 L-Hydrogel	2,39	3,81	1,74	0	2,88	1,79
% of live cells M2b	Static	M2 Plastic	1,1	1,21	0,55	1,68	0,7	0,54
% of live cells M2b	Static	M2 L-Scaffold	1,54	1,27	0,53	3,51	2,56	0,36
% of live cells M2b	Static	M2 L-Hydrogel	0,5	0,63	0,19	2,62	0,28	1,39
% of live cells M2b	Cyclic Stretch	M0 Plastic	0,87	1,57	0,77	1,54	2,11	0,88
% of live cells M2b	Cyclic Stretch	M0 L-Scaffold	0,6	0,84	0,66	2,39	0,63	0,31
% of live cells M2b	Cyclic Stretch	M0 L-Hydrogel	1,4	0,94	0,98	3,33	2,27	0
% of live cells M2b	Cyclic Stretch	M1 Plastic	1,62	1,95	2,69	1,17	0	3,36
% of live cells M2b	Cyclic Stretch	M1 L-Scaffold	2,52	1,55	1,86	1,22	1,04	1,09
% of live cells M2b	Cyclic Stretch	M1 L-Hydrogel	2,81	1,83	1,82	0,99	2,16	0
% of live cells M2b	Cyclic Stretch	M2 Plastic	1,11	1,98	0,96	1,61	2,03	0,34
% of live cells M2b	Cyclic Stretch	M2 L-Scaffold	1,12	1,08	0,22	3,82	2,24	1,42
% of live cells M2b	Cyclic Stretch	M2 L-Hydrogel	2,73	0,14	0,89	3	0,83	2,77
% of live cells M2c	Static	M0 Plastic	4,06	0,76	0,37	0,52	0,23	0,19
% of live cells M2c	Static	M0 L-Scaffold	0,5	0,97	0,46	0,9	0,067	0,18
% of live cells M2c	Static	M0 L-Hydrogel	0,71	0,41	0,51	0	0,57	0,053
% of live cells M2c	Static	M1 Plastic	0,96	1,8	0,58	0	0	0
% of live cells M2c	Static	M1 L-Scaffold	0,48	3,16	5,26	0	0	0
% of live cells M2c	Static	M1 L-Hydrogel	1,45	1,14	7,34	0	0	0

% of live cells M2c	Static	M2 Plastic	4,83	10,8	3,24	1,66	1,94	1,58
% of live cells M2c	Static	M2 L-Scaffold	24,3	9,57	6,44	1,61	2,76	1,34
% of live cells M2c	Static	M2 L-Hydrogel	9,69	1,48	5,47	1,84	1,48	1,9
% of live cells M2c	Cyclic Stretch	M0 Plastic	1,29	0,87	0,79	0,16	0,53	0,61
% of live cells M2c	Cyclic Stretch	M0 L-Scaffold	0,55	1,41	0,72	0,4	0,17	0,087
% of live cells M2c	Cyclic Stretch	M0 L-Hydrogel	0,7	0,6	1,45	0,18	0,97	0,75
% of live cells M2c	Cyclic Stretch	M1 Plastic	0,18	0,74	1,74	0	0	0
% of live cells M2c	Cyclic Stretch	M1 L-Scaffold	0,47	4,33	7,42	0	0,35	0
% of live cells M2c	Cyclic Stretch	M1 L-Hydrogel	1,83	0,61	3,51	0	0	0
% of live cells M2c	Cyclic Stretch	M2 Plastic	7,48	9,69	7,24	1,03	1,65	0,67
% of live cells M2c	Cyclic Stretch	M2 L-Scaffold	9,05	7,96	2,51	0,95	2,32	2,62
% of live cells M2c	Cyclic Stretch	M2 L-Hydrogel	6,5	2,09	10,7	1,02	1,59	3,17

Figure S2. Representative plastic conditions phase contrast images. A.Static B. Cyclic Stretch. Scale Bar 250 µm

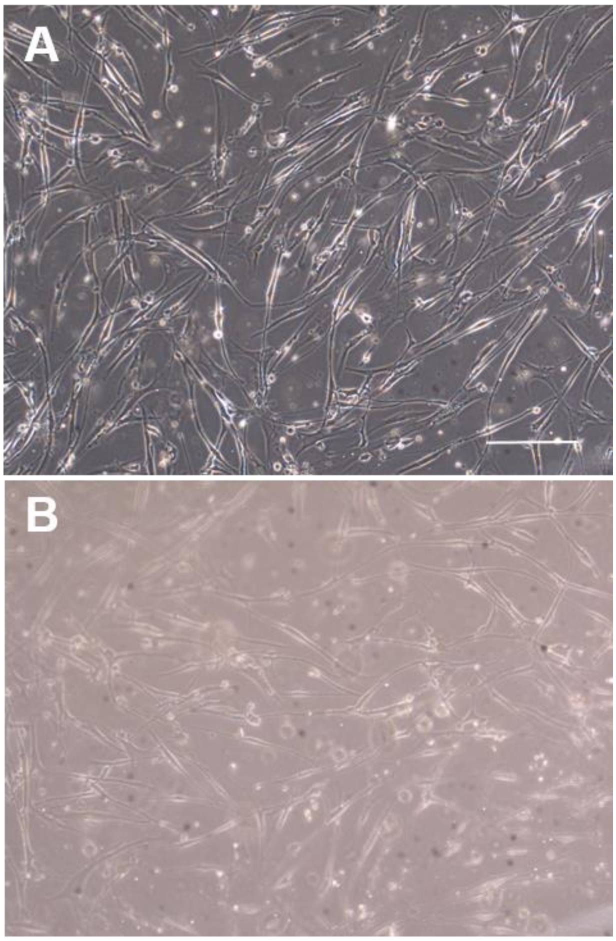

Supplement: Supplementary file 1 [file cells-11-01866-s001.zip › Supplement Material and methods v2.pdf]
