# Supplementary figures and images for: hLMSC Secretome Affects Macrophage Activity Differentially Depending on Lung-Mimetic Environments
Source: Cells. 2022 Jun 8;11(12):1866. doi: 10.3390/cells11121866 (PMC9221297; doi:10.3390/cells11121866)

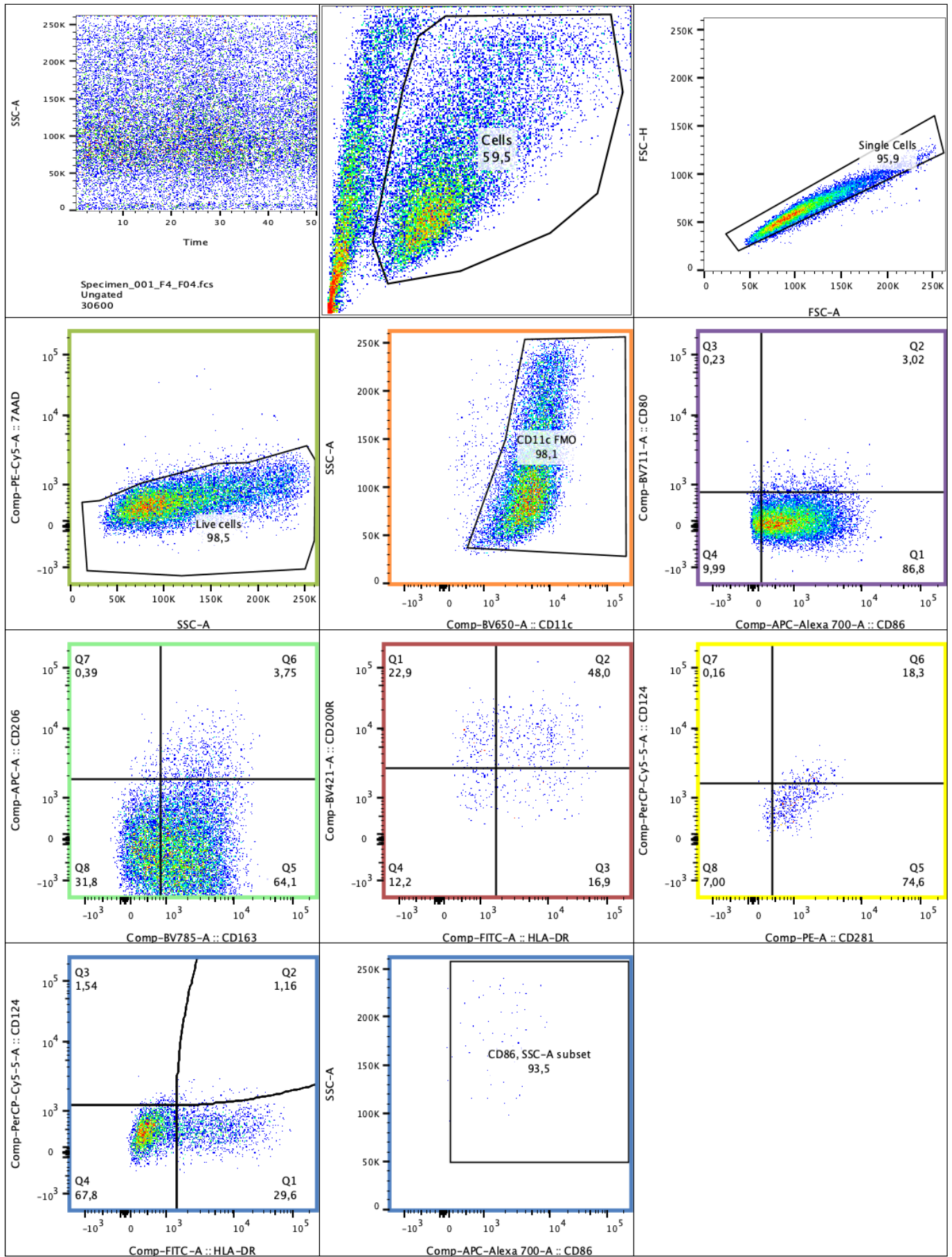

Supplement: Supplementary file 1 [file cells-11-01866-s001.zip › FACS gating strategy.pdf]

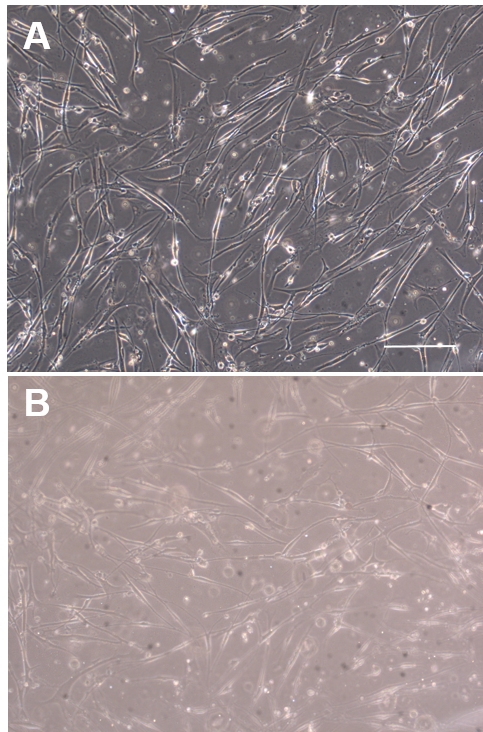

Supplement: Supplementary file 1 [file cells-11-01866-s001.zip › Figure S2.tif]
